# MonoNet: enhancing interpretability in neural networks via monotonic features

**DOI:** 10.1093/bioadv/vbad016

**Published:** 2023-02-23

**Authors:** An-Phi Nguyen, Dana Lea Moreno, Nicolas Le-Bel, María Rodríguez Martínez

**Affiliations:** Department of Mathematics, ETH Zürich, Zürich 8092, Switzerland; Cognitive Health Care and Life Sciences, IBM Research Europe, Zürich 8803, Switzerland; Cognitive Health Care and Life Sciences, IBM Research Europe, Zürich 8803, Switzerland; Department of Mathematics, EPFL, 1015 Lausanne, Switzerland; Department of Physics, ETH Zürich, Zürich 8092, Switzerland; Cognitive Health Care and Life Sciences, IBM Research Europe, Zürich 8803, Switzerland

## Abstract

**Motivation:**

Being able to interpret and explain the predictions made by a machine learning model is of fundamental importance. Unfortunately, a trade-off between accuracy and interpretability is often observed. As a result, the interest in developing more transparent yet powerful models has grown considerably over the past few years. Interpretable models are especially needed in high-stake scenarios, such as computational biology and medical informatics, where erroneous or biased models’ predictions can have deleterious consequences for a patient. Furthermore, understanding the inner workings of a model can help increase the trust in the model.

**Results:**

We introduce a novel structurally constrained neural network, *MonoNet*, which is more transparent, while still retaining the same learning capabilities of traditional neural models. MonoNet contains *monotonically* connected layers that ensure monotonic relationships between (high-level) features and outputs. We show how, by leveraging the monotonic constraint in conjunction with other *post hoc* strategies, we can interpret our model. To demonstrate our model’s capabilities, we train MonoNet to classify cellular populations in a single-cell proteomic dataset. We also demonstrate MonoNet’s performance in other benchmark datasets in different domains, including non-biological applications (in the Supplementary Material). Our experiments show how our model can achieve good performance, while providing at the same time useful biological insights about the most important biomarkers. We finally carry out an information-theoretical analysis to show how the monotonic constraint actively contributes to the learning process of the model.

**Availability and implementation:**

Code and sample data are available at https://github.com/phineasng/mononet.

**Supplementary information:**

Supplementary data are available at *Bioinformatics Advances* online.

## 1 Introduction

In recent years, state-of-the-art deep-learning networks have achieved outstanding predictive power in many disciplines. In computational biology, deep neural networks have found success in long-standing problems, such as the prediction of protein structure (Jumper *et al.*, 2021), variant calling (Poplin *et al.*, 2018), drug sensitivity prediction (Manica *et al.*, 2019), or *de-novo* compound design (Born *et al.*, 2021; Vamathevan *et al.*, 2019), just to mention a few applications. However, the gain in accuracy has often come at the price of transparency, with most models behaving as black-boxes and producing decisions that lack *interpretability*.

Being able to interpret the predictions of a machine learning model is of fundamental importance, especially in sensitive domains, such as healthcare, crime recidivism, or finance. If users do not trust a model, they will not use it, or even worse, they will use it and be inadvertently exposed to hidden biases (Lipton, 2018; Wachinger *et al.*, 2018; Wexler, 2018). On the other hand, if the system can explain its reasoning, then the soundness of the reasoning can be examined (Doshi-Velez and Kim, 2017).

Many efforts have been devoted in the last years toward the building of interpretable models that can provide information about how a decision was made. Interpretable methods can be broadly categorized in two groups: *ante hoc* models and *post hoc* methods. *Ante hoc* interpretable models are models that are interpretable by construction (Angelino *et al.*, 2017; Melis and Jaakkola, 2018; Yang *et al.*, 2017). Traditional examples of such models are decision trees (Quinlan, 1986), rule lists (Letham *et al.*, 2015) or risk score models (Ustun and Rudin, 2019). *Post hoc* interpretable models, on the other hand, aim at providing a way to explain a black-box model *after* it has been trained (Ancona *et al.*, 2018; Bach *et al.*, 2015; Lundberg and Lee, 2017; Ribeiro *et al.*, 2016, 2018; Selvaraju *et al.*, 2017; Shrikumar *et al.*, 2017; Simonyan *et al.*, 2013; Sundararajan *et al.*, 2017). Typical examples of *post hoc* methods are surrogate methods, such as LIME (Ribeiro *et al.*, 2016) and Anchors (Ribeiro *et al.*, 2018), or backpropagation-based attribution methods (Ancona *et al.*, 2018; Shrikumar *et al.*, 2017; Sundararajan *et al.*, 2017).

While *ante hoc* interpretability is preferable (Rudin, 2019), in practice, adding interpretability constraints into the model can be too restrictive and potentially impact its performance. Furthermore, an *ante hoc* model may quickly grow too complex, e.g. a very deep decision tree, and effectively become difficult to interpret *despite its transparency*. Conversely, *post hoc* methods tend to give only approximate insights into a model’s decision process, i.e. they may not faithfully describe the whole model’s functioning, but only its behavior in a subset of samples.

### 1.1 Motivation

**Fig. 1.**
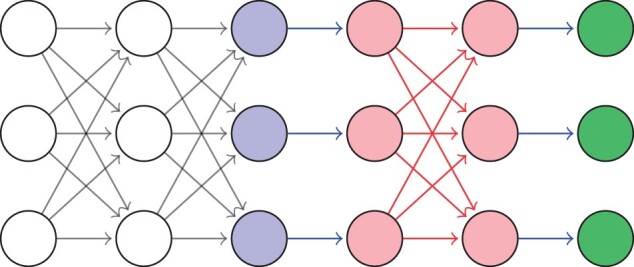
Visualization of a MonoNet. The interpretable layer (blue, 3rd layer from the left in the figure) is free to learn any representation of the previous layers (white, 1st and 2nd from the left). The pink layers (5th and 6th layers from the left) are guaranteed to be monotonically increasing with respect to each other because they are connected by means of positive weights (red arrows). Thanks to component-wise rescaling (blue arrows), the output (green, right-most layer) can have any *arbitrary* monotonic relationship with respect to the interpretable layer. The whole architecture is explained in Section 2.2

In this article, we propose a compromise solution between *ante hoc* and *post hoc* methods. We introduce a special class of neural networks, which we call *MonoNets*, aiming at increasing transparency and interpretability by imposing *monotonic constraints* between a hidden layer and the output prediction. Briefly, in a MonoNet, the layers from the input to a chosen hidden layer (say *k*) are left unconstrained, while the layers from *k* to the output are built to enforce a monotonic relationship (defined in Section 2.1) between the layer *k* and the output layer. This construction allows the layer *k* to learn arbitrary high-level features, which we can attempt to understand in a *post hoc* fashion. The *ante hoc* monotonic constraints will then readily allow us to interpret the output in terms of the high-level features. Figure 1 graphically summarizes the architecture of a MonoNet. The idea is that by imposing constraints only on one part of the network, this transparent/interpretable part of the model will not grow too complex, which will allow us to more easily interpret the predictions in terms of the high-level features. Furthermore, we will be able to retain the learning/approximation capabilities of classical neural networks (Section 2). Finally, by using a *post hoc* method *only on the unconstrained part* of the model, we expect to get more easily faithful *post hoc* explanations.

**Why monotonicity?** At this point, a question may arise: how does monotonicity enhance interpretability? Our argument is based on the comparison between linear and non-linear classifiers. Arguably, linear classifiers are regarded as interpretable because users can trivially understand if an output *increases* or *decreases* when a predictor is changed, independently from the other predictors. On the other hand, in non-linear classifiers, the possible correlations among input variables make it difficult to predict how the output changes following a single variable change. Enforcing monotonicity allows us to relax the linearity constraint, while still being able to reason about the behavior of the output with respect to a single predictor independently of the others. This will grant us a certain degree of interpretability about the model predictions.

### 1.2 Related work

Our work inserts itself in a recent line of interpretability research focused on *structurally constrained* networks. Neural Additive Models (Agarwal *et al.*, 2021) process *each* input feature with a *distinct* neural network. The outputs of each of these networks are then summed up to obtain the final prediction. We argue that this imposes too strong a constraint, and may impact the model performance on more complex tasks. In Section 2, we will discuss how MonoNets can retain the same approximation capabilities of unconstrained deep networks. Perhaps the models most closely related to MonoNets are Self-Explaining Neural Networks (SENNs) (Melis and Jaakkola, 2018). Similarly to our work, SENN’s architecture first learns high-level features that are then monotonically related to the output. However, this monotonic relation is restricted to being *locally* and *additively* separable. That is, for each sample, they can compute an *importance* score to understand which high-level feature plays a more important role in a prediction. While MonoNets do not provide an importance score, they do provide an understanding of how a high-level feature impacts an output, in the sense that if an output monotonically increases or decreases with respect to the feature. Furthermore, this behavior is global, i.e. valid for *all* the samples, and the model does *not* need to be interpreted one sample at a time.

## 2 Methods: building a MonoNet

### 2.1 Notation and definitions

We represent vectors with lowercase boldface letters, e.g. x, and matrices with uppercase boldface letters, e.g. W. Elements of vectors and matrices are denoted with lowercase subscripts. The *i*-th element of vector x is $x_{i}$ and the element in row *i* and column *j* of matrix W is $W_{ij}$. Given a function y=f(x), we denote by $\frac{\partial y_{i}}{\partial x_{j}}$ the partial derivative of the *i*-th component of y with respect to the *j*-th component of x.

In this work, we focus on multilayer perceptrons (MLPs). Let the function y=f(x) implement an MLP with L+1 layers. We denote a layer *k* of the network by $h^{\left(k\right)}$ for k=0,…,L. In particular, the input x and the output y correspond, respectively, to $h^{\left(0\right)}$ and $h^{\left(L\right)}$. As is customary, we represent a non-linearity as σ(⋅). We can therefore write $h^{\left(k+1\right)}=\sigma (W^{\left(k\right)}h^{\left(k\right)}+b^{\left(k\right)})$, where $W^{\left(k\right)}$ is the weight matrix and $b^{\left(k\right)}$ is the bias.

Since there are multiple ways of defining an order in $R^{n}$, we shall clarify which notion of monotonicity we are working with.

Definition 2.1. A function $f:R^{n}\to R$ is called *monotonically increasing* (or non-decreasing) if for all i=1,…,n the (univariate) restriction $f{|}_{i}:x_{i}\mapsto y=f({\tilde{x}}_{1},\ldots ,x_{i},\ldots ,{\tilde{x}}_{n})$, obtained by fixing all the components except the *i*-th, is monotonically increasing for every fixed value ${\tilde{x}}_{j}$∀j=1,…,n and j≠i, in the usual sense of monotonicity for univariate functions. The definition for monotonically decreasing functions is analogous.

Definition 2.2 A multivalued function $f:R^{n}\to R^{m}$ is called *monotonically increasing* if every component $f_{i}$ with i=1,…,m is monotonic according to Definition 2.1.

### 2.2 Monotonic features

Let us assume we want to interpret the prediction of an MLP with L+1 layers with respect to its *k*-th layer. In this section, we present a way to constrain the $N^{\left(k\right)}$ units of the chosen interpretable layer $h^{\left(k\right)}\in R^{N^{\left(k\right)}}$ to be monotonic with respect to the units of the output layer $y\in R^{N^{\left(L\right)}}$. As mentioned before, in this work, we focus only on MLPs. However, our strategy can be extended to different architectures (Supplementary section ‘Extending MonoNets to other architectures’).

#### 2.2.1 Monotonically increasing layers

The first step in our construction is building monotonically increasing layers. We follow the same idea as in Daniels and Velikova (2010) and Sill (1998). As discussed in Section 2.1, the $h^{\left(k+1\right)}$ layer can be computed from the elements of layer $h^{\left(k\right)}$ as $h^{\left(k+1\right)}=\sigma (W^{\left(k\right)}h^{\left(k\right)}+b^{\left(k\right)})$. We can now compute the partial derivatives of this relationship as:


(1) $$\frac{\partial h_{i}^{\left(k+1\right)}}{\partial h_{j}^{\left(k\right)}}=\underset{\geq 0}{\underset{︸}{\sigma^{\prime }(\sum_{t=1}^{N^{\left(k\right)}}W_{it}^{\left(k\right)}h_{t}^{\left(k\right)}+b_{i}^{\left(k\right)})}}W_{ij}^{\left(k\right)}.$$

The most commonly used non-linearities are non-decreasing functions, whose derivatives are always non-negative. Hence, the partial derivative in (1) will be non-negative if and only if $W_{ij}^{\left(k\right)}\geq 0$. That is, $h^{\left(k+1\right)}$ will be monotonically non-decreasing with respect to $h^{\left(k\right)}$ if and only if the weight matrix has only non-negative entries. A way to impose this constraint is to apply to the weights a function with range in the positive numbers, e.g. the element-wise exponential function:


(2) $$h^{\left(k+1\right)}=\sigma (\text{exp}(W^{\left(k\right)})h^{\left(k\right)}+b^{\left(k\right)}).$$

Other functions are possible, such as the (element-wise) square function $f(W)=W^{2}$ or the translated hyperbolic tangent f(W)=tanh(W)+1.

Since the compositions of monotonically non-decreasing functions are also monotonically non-decreasing, we are guaranteed that, by stacking such layers, the last layer $y=h^{\left(L\right)}$ is monotonically non-decreasing with respect to the interpretable layer $h^{\left(k\right)}$.

#### 2.2.2 Allowing arbitrary monotonicity

The construction in the previous section enabled us to ensure a monotonically non-decreasing relationship between a chosen interpretable layer and the output. However, we would like *each* component of the interpretable layer $h^{\left(k\right)}$ to have an *arbitrary* monotonic behavior (i.e. either increasing or decreasing) with respect to *each* component of the output y. This can be achieved by component-wise rescaling of both $h^{\left(k\right)}$ and y. To see this let us introduce the auxiliary layers ${\tilde{h}}^{\left(k\right)}$ and $\tilde{y}$ so that where $\alpha \in R^{N^{\left(k\right)}}$, $\beta \in R^{N^{\left(L\right)}}$ and ⊙ denote a component-wise multiplication. If we stack monotonic layers from ${\tilde{h}}^{\left(k\right)}$ to $\tilde{y}$ as explained in Section 2.2.1, we find that the partial derivatives are where the partial derivatives in terms of the auxiliary layers are positive.


(3) $${\tilde{h}}^{\left(k\right)}=\alpha ⊙h^{\left(k\right)},\;\;y=\beta ⊙\tilde{y},$$

(4) $$\frac{\partial h_{i}}{\partial y_{j}}=\underset{\geq 0}{\underset{︸}{\frac{\partial {\tilde{h}}_{i}}{\partial {\tilde{y}}_{j}}}}\frac{1}{\alpha_{i}\beta_{j}},$$

#### 2.2.3 Approximation capabilities of MonoNets

Daniels and Velikova (2010) proved an analogue of the universal approximation theorem (Hornik, 1991) for monotonically non-decreasing functions. It has to be noted that the definition of monotonicity used by Daniels and Velikova (2010) is more relaxed than ours. Therefore, their theorem is valid in particular for our definition of monotonicity (Definition 2.1). Since any monotonically non-increasing function can be obtained by changing the sign of a monotonically non-decreasing function, the result can be extended to monotonically non-increasing functions. Now the question is, given a MonoNet with L+1 layers, do we still retain the same universal approximation capabilities of neural networks by constraining the output layer y to be monotonic with respect to $h^{\left(k\right)}$? The answer is yes. This can be understood with a somewhat extreme example. The last layer of deep-learning architectures is always monotonic (either linear or with known non-linearity given by the activation function) according to Definition 2.2, and hence can be potentially approximated by our monotonic construction. This means that the apparently constrained MonoNet can in fact approximate any function that a classic (unconstrained) neural network that has the *same* first k−1 layers can approximate. However, this is not the use case of interest. Instead, our model becomes useful when an inference problem can be solved by learning a hidden representation that has an *arbitrary non-linear monotonic* relationship with respect to the output.

#### 2.2.4 Interpreting the unconstrained block

While we argued that monotonicity can improve our understanding of a model, to attain full interpretability of a MonoNet we need to be able to interpret the high-level features with respect to the input features. Any *post hoc* strategy can be used toward this end. In Section 3.2.2, we show this in three different ways: by simple statistical analysis, using causal methods and using feature attribution methods.

## 3 Results

### 3.1 Performance benchmarking

We benchmark MonoNet on a series of tabular datasets, as well as (biomedical) image datasets. Among the tabular datasets, we consider datasets provided by the UCI Machine Learning Repository (Dua and Graff, 2017), as well as a single-cell proteomics dataset (Levine *et al.*, 2015). Since these datasets are relatively simple, we do not compare MonoNet to other unconstrained deep models, but limit the comparison to two interpretable models, risk-slim (Ustun and Rudin, 2017) and decision trees (Quinlan, 1986). In the vision domain, we test our model on the biomedical image datasets provided by Yang *et al.* (2021, 2023). Since the more traditional machine learning models are not directly scalable to this domain, we compare MonoNet to established unconstrained neural network models [i.e. ResNet (He *et al.*, 2016)].

We report the results on tabular and image datasets in Tables 1 and 2. It can be observed that MonoNet performance is comparable to established interpretable models in tabular datasets. Therefore, in these cases, it may not be necessary to use a MonoNet, given the extra effort needed to interpret it (Section 3.2.2). On the other hand, as the tasks become more complex, classical interpretable methods may not be applicable. Hence, MonoNet may be an acceptable compromise between accuracy and interpretability. While this is clearer in the image datasets (Table 2), this trade-off can already be observed on a smaller scale in the single-cell dataset (Table 1). Indeed, an unconstrained decision tree achieves almost perfect accuracy on this dataset. However, since this tree has depth 17, it is effectively *difficult to interpret*, despite its transparency. To maintain interpretability, either a regularized model (e.g. a tree with depth constrained to be <5) or a MonoNet can be trained: the two models achieve comparable results.

**Table 1.** Test accuracies (in %) on risk score predictions (Ustun and Rudin, 2019), as well as the single-cell dataset by Levine *et al.* (2015)

Model	Dataset					
	Income	Mammo	Mushroom	Breast	Bank	Single cell
risk-slim	75.31	53.61	**100.00**	84.06	61.30	—^a^
Decision Tree	82.16	**76.29**	96.92	94.20	57.49	88.85 (**99.59**)^b^
MonoNet	**84.29**	71.65	96.01	**95.79**	**63.05**	90.2

*Note*: We compare our model against two interpretable models: risk-slim (Ustun and Rudin, 2019), and decision trees (Quinlan, 1986). The best results are reported in bold characters.

a risk-slim is applicable only on binary features and, therefore, is omitted from the single-cell dataset benchmarking.

b In this table, we report the results obtained for a tree constrained to depth 5, together with the result obtained by an unconstrained tree (in parentheses): the unconstrained tree has depth 17 and therefore is *difficult to interpret*.

**Table 2.** Test accuracies (in %) on (down-sampled) biomedical imaging datasets as provided by Yang *et al.* (2021, 2023)

Model	Dataset					
	PathMNIST	BreastMNIST	BloodMNIST	DermaMNIST	PneumoniaMNIST	OrganAMNIST
ResNet18	90.7	**86.3**	95.8	73.5	85.4	**93.5**
ResNet50	**91.1**	81.2	95.6	73.5	85.4	**93.5**
auto-sklearn	71.6	80.3	87.8	71.9	85.5	76.2
AutoKeras	83.4	83.1	96.1	74.9	87.8	90.5
Google AutoML Vision	72.8	86.1	**96.6**	**76.8**	**94.6**	88.6
MonoNet	83.6	81.4	88.1	73.3	81.6	79.5

*Note*: We compare our monotonic CNN in Figure 6 to established state-of-the-art CNN models, i.e. ResNets (He *et al.*, 2016), as well as automatically optimized models as provided by auto-sklearn (Feurer *et al.*, 2015), AutoKeras (Jin *et al.*, 2019) and Google AutoML Vision. All results, apart from MonoNets, are taken from Yang *et al.* (2023). Best performing model on each dataset are indicated with bold face.

### 3.2 Interpretability analysis for single-cell classification

In this section, we illustrate how to interpret a MonoNet. As a case study, we choose the relatively simple task of cell classification from single-cell proteomics data (Levine *et al.*, 2015). In the next section, we will show one way to extend this interpretability analysis to the vision domain.

#### 3.2.1 Dataset and model details

We analyze the single-cell proteomics dataset provided by Levine *et al.* (2015). The data consist of 81 075 single cells from 20 immune subpopulations. The abundance of 13 cell surface markers was measured by mass cytometry. The markers and cellular populations are summarized in Supplementary Table S3.

For training, we split the dataset into a training (80%) and a test (20%). To monitor the learning of the model during training, we retain (10%) of the training set as a validation set.

To solve the task, we design a MonoNet consisting of the following layers:

- the initial input layer of size 13;
- three unconstrained layers of size, respectively, 16, 16 and 8. The layer of size 8 will constitute the interpretable layer, i.e. the layers whose neurons can be interpreted because monotonically constrained with respect to the output neurons;
- a point-wise multiplication layer as the one shown in (3) that will allow for arbitrary monotonic direction. In the following, we will refer to this layer as ‘Pre-monotonic’;
- two monotonic layers of size 32; and
- a final output layer of size 20.

This is the same model whose performance is reported in Table 1.

#### 3.2.2 Interpreting the unconstrained block

In a MonoNet, the monotonic block is *transparent*, and therefore, it is straightforward to inspect the effect of a neuron in the interpretable layer on an output class, i.e. the relationship is either monotonically increasing or decreasing. However, to fully interpret the model, we need to first understand what are the high-level features encoded in the interpretable layer. In this section, we show three possible ways to extract these high-level features in terms of the original input biomarkers.

**Fig. 2.**
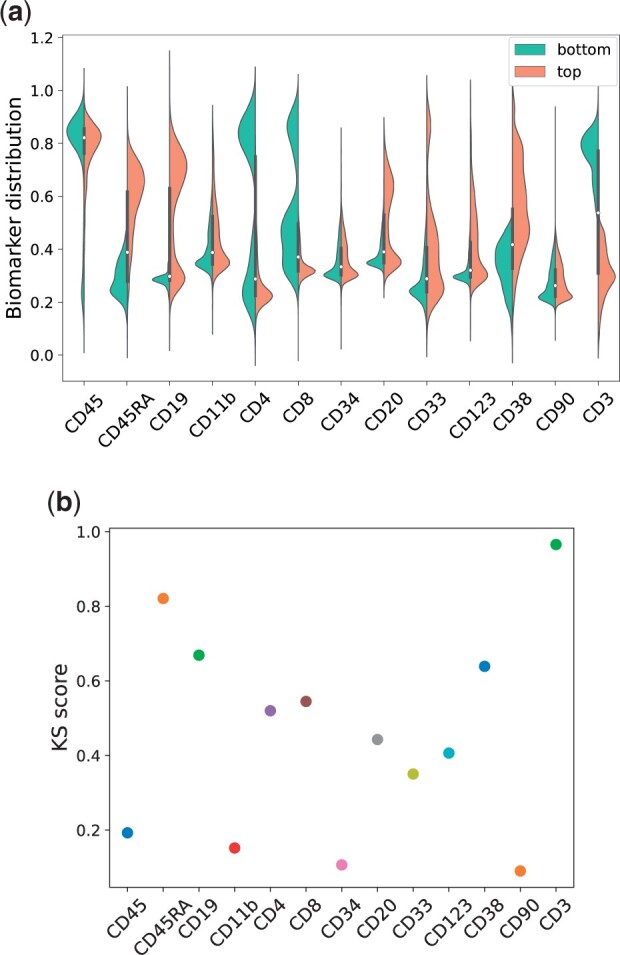
(**a**) Violin plot showing the top and bottom distributions of the biomarkers for the fourth interpretable neuron and (**b**) the corresponding KS scores also for Neuron 4. This particular interpretable neuron shows a specialization to recognize markers CD3, CD45RA, CD19 and CD38

**(1) Statistical analysis of the activation patterns.** A first possible approach is to carry out an exploratory analysis of how the input biomarkers affect each neuron of the interpretable layer. For each neuron, we rank single cells according to how much they activate that neuron. Next, we select the top 20% and bottom 20% single cells in the activation rank and compare the biomarker distribution in both subsets. The idea is that we should be able to identify neurons that have specialized to recognize a given biomarker by comparing the biomarkers distributions in the top and the bottom activating cells. As a visual example, for the fourth neuron of the interpretable layer, we plot in Figure 2a, the distribution of the input features in the top and bottom activating sets as side-by-side violin plots. It can be noted that the chosen neuron seems to differentiate several markers, such as CD3, CD45RA and CD38, as demonstrated by the presence of two well-separated distributions. Furthermore, this figure can also suggest the direction of the relationship between a biomarker and its corresponding neuron activation. For instance, the neuron is more activated by lower values of CD3 and high values of CD45RA, respectively.

We observe bimodal distributions in a few cases, e.g. the distribution of CD19 in the top activating cells (red distribution) or CD4 and CD8 in the bottom activating cells (green distribution). This bi-modality is likely to arise from the presence of a given marker in several communities, e.g. CD19 is ubiquitously expressed throughout all stages of B-cell differentiation, and CD4 and CD8 are expressed in both naive and mature CD4${}^{+}$ and CD8${}^{+}$ T cells, respectively (Supplementary Table S3).

Visual inspection cannot be done extensively for all neurons and markers. We, therefore, compute the two-sample Kolmogorov–Smirnov (KS) score (Ross, 2004) to more quantitatively measure the difference between the distributions of top and bottom activating cells. Briefly, given two samples $X_{1}$ and $X_{2}$, respectively, of size $m_{1}$ and $m_{2}$ drawn from continuous distributions, we can compute their empirical cumulative density functions as follows:


(5) $$\mathrm{ECD}F_{X_{i}}(x)=\frac{\#\text{elements in}\;X_{i}\leq x}{m_{i}},\;\text{with}\;i\in \{1,2\}.$$

The KS score is then defined as the maximum distance between the two empirical cumulative density functions $D_{m,n}=\text{max}|\mathrm{ECD}F_{X_{1}}(x)-\mathrm{ECD}F_{X_{2}}(x)|$. Figure 2b shows the KS scores for the fourth neuron and confirm the intuition we gained by inspecting Figure 2a. Numerical results for all biomarkers and neurons are given in Supplementary Table S4.

To assess the *global* importance of the biomarkers on the classification task (i.e. to assess which biomarkers are generally the most informative for the task), we can average the KS scores across all interpretable neurons (Supplementary Fig. S11a). For example, CD90 has clearly a very low mean KS score across all the interpretable neurons, meaning that it is possibly never used to perform a prediction.

We note that, while the threshold to define top and bottom distribution may seem arbitrary, the ‘most different differentiated biomarkers’ for each interpretable neuron tend to be robust to the choice of the threshold. This is shown in Supplementary Figures S15–S22. The higher the threshold, the lower the KS score tends to be. However, if a biomarker is well separated at lower thresholds (e.g. CD3 or CD11b in Supplementary Fig. S16), it remains ‘more differentiated’ *relatively to other markers* also at higher thresholds.

**Fig. 3.**
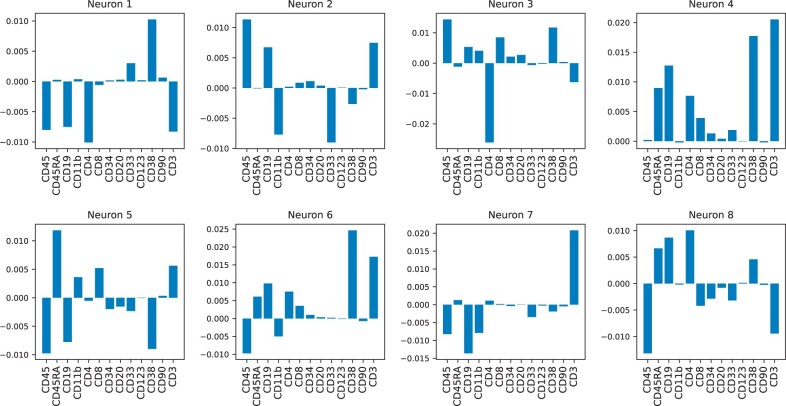
Shapley values computed with KernelShap for all interpretable neurons. The plot shows that neurons in the interpretable layer specialize in the recognition of only a few markers. We also observe a general agreement between the neuron–markers associations identified using the KS score and Shapley values

**(2) Feature attribution methods.** Another way of assessing the link between the input biomarkers and the interpretable layer is to use feature attribution methods (Ancona *et al.*, 2018). Feature attribution methods can be thought of as sensitivity analysis methods: for a given output of interest (in our case, the activation value of the interpretable neurons), an attribution method computes how much a change in an input will affect the output. One way to compute these attributions is Shapley values (Lundberg and Lee, 2017). The results are reported in Figure 3. The figure shows that each neuron specializes in the recognition of just a few markers, as most neurons show few peaks.

We observe a general agreement with the results from the KS score analysis. For Neuron 4, this can be qualitatively observed by comparing the violin plots in Figure 2a and the Shapley values in the top rightmost plot of Figure 3. Indeed, for Neuron 4, the four highest KS scores are obtained (in decreasing order) for CD3, CD45RA, CD19 and CD38 (numerical values can be inspected in Supplementary Table S4). The four highest Shapley values are given to CD3, CD38, CD19 and CD45RA. In the Supplementary Material, we show similar results using other feature attribution methods, such as Integrated gradients (Sundararajan *et al.*, 2017) and Neuron Conductance (Dhamdhere *et al.*, 2019).

Also on a global level, the average KS scores (Supplementary Fig. S11a) and the average Shapley values (Supplementary Fig. S11b) show some agreement. It can be qualitatively seen from the plots that both strategies are mostly in agreement. For instance, both methods give low scores to markers CD90 and CD34. Similarly, markers CD3, CD45 and CD4, are identified by both methods as highly relevant for the classification task. We do not expect a perfect agreement (e.g. regarding the importance of CD20) since both methods rely on some heuristics/approximation (e.g. the 20% threshold in selecting top and bottom distribution for the KS score, and the approximate nature of Shapley values).

**Fig. 4.**
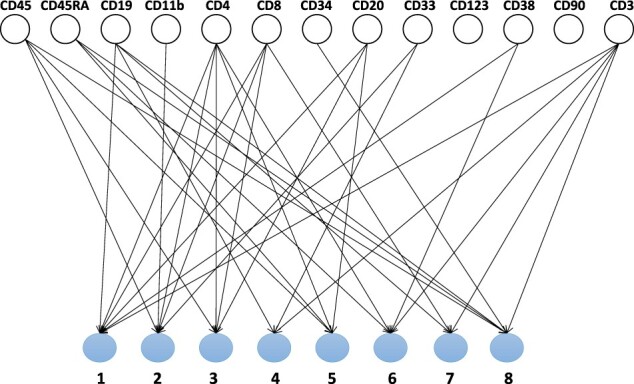
Causal structure inferred using kPC algorithm on the input biomarkers and the interpretable neurons. Similarly to Supplementary Figure S11b, the PC algorithm assigns low predictive values to markers CD90 and CD123

**Causal models for *post hoc* interpretability.** Inspired by Sani *et al.* (2021), we explore the application of causal discovery methods to get insights about the relationship between the input biomarkers and the interpretable neurons. We apply the PC algorithm (Spirtes *et al.*, 2000) to infer the causal relationship between the biomarkers and the interpretable neurons via conditional independence tests. In particular, we make use of the kernel-based implementation (kPC) by Zhang *et al.* (2012). The inferred causal structure is shown in Figure 4. We find a good (but, again, not perfect) agreement between the results of the PC algorithm and our previous analyses: Neuron 4 is predicted to be causally influenced by CD3, CD33 and CD19, which means that all three methods agree on two markers, CD3 and CD19.

#### 3.2.3 Interpreting the monotonic block

**Fig. 5.**
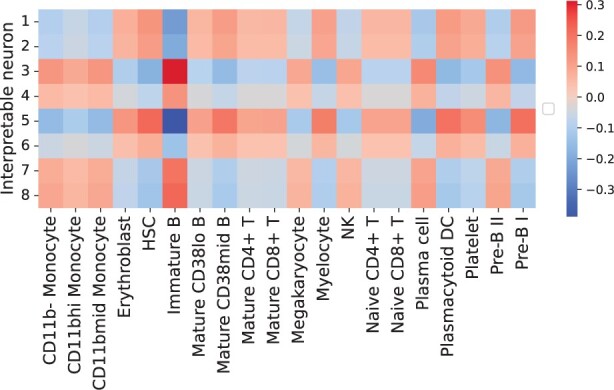
Correlation matrix between the interpretable neurons and the y labels (cell types). This matrix gives us information about the type of monotonic relationship (negative or positive) between each interpretable neuron and each predicted class

Having characterized the high-level features in terms of the input biomarkers, completing the interpretation of the model is straightforward. By construction, every neuron in the interpretable layer has a monotonic relationship with respect to each output neuron of the model. For each pair of interpretable neurons and output class, we can easily inspect the direction/strength of their relationship by computing $\frac{1}{\alpha_{i}\beta_{j}}$ [c.f. Section 2.2.2 and Equation (4)]. We show this matrix in Figure 5.

We can now put together the information given by the matrix in Figure 5 and the information extracted in the previous analyses of the unconstrained block. To continue with the example on Neuron 4 from the previous section (Fig. 2b), we know that Neuron 4 is mostly activated when CD19 and CD38 have higher values. If we now look at the matrix in Figure 5, we notice that, e.g. immature B cells are positively correlated with Neuron 4, i.e. the higher the value for Neuron 4, the higher the chance that MonoNet will predict the sample as immature B cell. Putting everything together, according to MonoNet, immature B cells are then characterized by high values of CD38 and CD19: this is an accordance with our expectations from known biology (Levine *et al.*, 2015), as reported in Supplementary Table S3.

### 3.3 Interpretability analysis for colorectal cancer classification

In this section, we briefly show how a similar interpretability analysis can be carried out for a Convolutional Neural Network (LeCun *et al.*, 1998). The most straightforward way to extend our construction to convolutional layers is to impose the same constraint Equation (2) to the weights of the convolutional filters. We argue however that this may not be necessary for the first layer of convolutional filters. Indeed, this first layer is usually easily interpreted as a feature detector (e.g. edge detector) (Zeiler and Fergus, 2014). However, the monotonicity constraint may be necessary starting from the second convolutional layer as more complex non-linear interactions between features start to appear [as shown by Zeiler and Fergus (2014)].

**Fig. 6.**
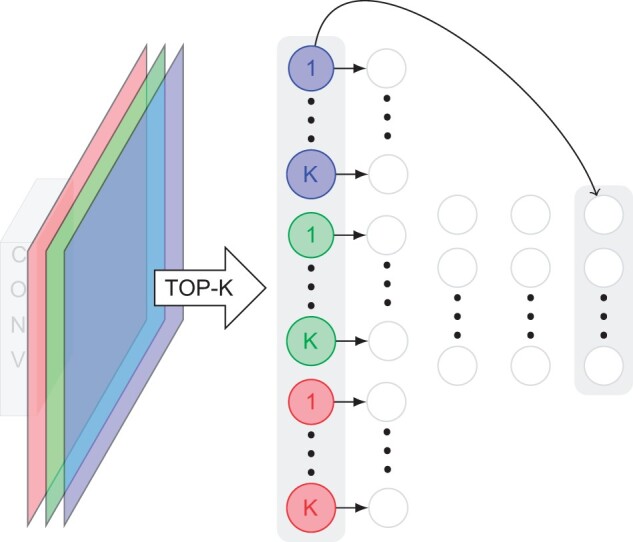
Monotonic CNN. Using an (unconstrained) convolutional layer, we generate different activation maps (colored planes). For each activation map, we compute and extract the K most activated locations (colored circles). We then enforce a monotonic relationship between these most activated units and the output. This relationship is implemented by summing a monotonic block together with a monotonic residual connection (curved arrow). Note that the sum of two monotonically increasing (resp. decreasing) functions is also monotonically increasing (resp. decreasing)

In our experiments (Table 2), a second layer of convolutions did not improve the results. Instead, we simply use a single convolutional layer to extract features. From each feature map, we then select only the top-*k* activations (with *k* varying across the datasets). We use these top-*k* features as interpretable neurons and impose on them a monotonic relationship with respect to the output predictions. In particular, this monotonic relationship is implemented using a monotonic block of two monotonic layers (similarly to the tabular case) summed together with a *monotonic residual connection*, i.e. a single monotonic layer that directly connects the interpretable layer to the output layer. A visual schematic is shown in Figure 6. This construction allowed us to achieve similar results to ResNets (Table 2).

**Fig. 7.**
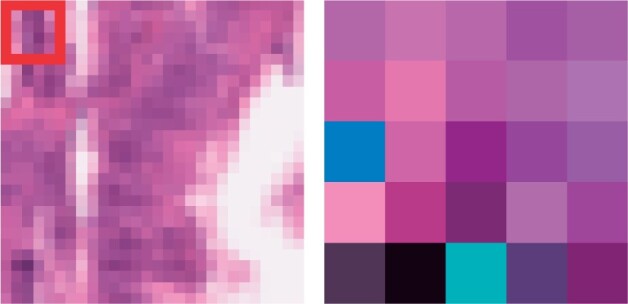
Interpreting a monotonic CNN trained on PathMNIST (Kather *et al.*, 2019). Here, we visualize the sample (left) and a high-level feature (right) that we want to interpret, i.e. one of the convolutional filters learned by the model. By construction, we know where this feature is ‘maximally detected’ in the original image (red square). By analyzing the monotonic block, we also know that this feature is positively correlated to the class ‘colorectal adenocarcinoma epithelium’. Overall, we can conclude that the top left corner of the image provides supporting evidence for the class

Such a monotonic CNN can be interpreted in a two-step approach, similarly to the tabular case (Section 3.2). In this case, the unconstrained block is constituted by the convolutional layer, followed by the top-*k* activations extraction. One simple way to interpret this part is to first visualize the convolutional filters and then locate their maximal activations on the image. Then, the interpretation of the monotonic block is as easy as in the tabular case: by looking at the coefficient $\frac{1}{\alpha_{i}\beta_{j}}$ [cf. Equation (4)], we can infer the directionality and strength of the monotonic relationship. As a concrete example, we analyze a Monotonic CNN trained on PathMNIST (Yang *et al.*, 2023), a down-sampled version of the histological dataset provided by Kather *et al.* (2019) to study colorectal cancer. Figure 7 shows 1 of the 256 convolutional filters (right) and its location on the sample we are interpreting (left). If we focus on the class predicted by the network, *colorectal adenocarcinoma epithelium*, the positivity of the coefficient $\frac{1}{\alpha_{i}\beta_{j}}$ tells us that the feature extracted by this particular filter is *monotonically increasing* with respect to that class. Putting everything together, in this example the monotonic CNN finds in the left-top corner of the image some evidence for the class *colorectal adenocarcinoma epithelium*. Note that if the dataset was of higher resolution, and the convolutional filters were bigger, it may have been possible to assign some ‘histopathological significance’ to the filter, further strengthening the interpretation of the network.

### 3.4 Where does the classification happen?

In the previous sections, we have seen how monotonicity can be leveraged to make complex deep models more interpretable. However, an interesting question would be if the monotonic block contributes at all toward the classification, or is the classification mostly performed by the unconstrained block? In the latter case, the interpretation of the monotonic block would not be very informative of the decision process of MonoNet (since most of the decision would be taking place in the unconstrained block). In this section, we attempt to answer this question in two ways: by analyzing the information flow across the layers, and by analyzing the activation patterns of the layers. For these analyses, we consider again the MonoNet trained on the single-cell classification task (Section 3.2).

#### 3.4.1 Monitoring the information flow across layers

**Fig. 8.**
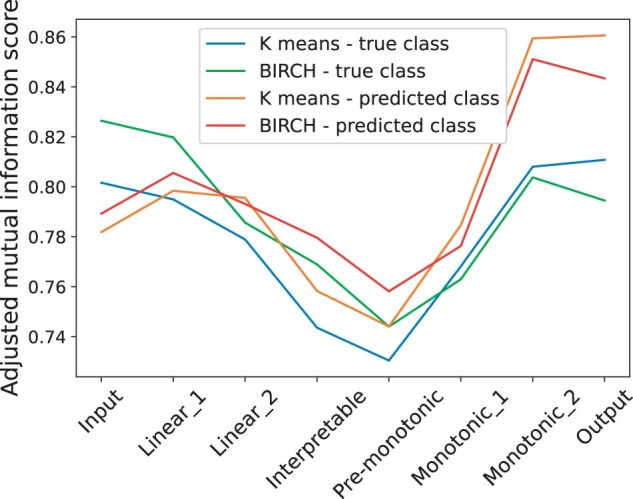
AMI between each layer clustering and class labels. For each layer, we run two clustering algorithms (BIRCH and K-means) on the activation values of all test samples in the corresponding layer and use AMI to compare this clustering with respect to the predicted or true class of the samples

Following a similar strategy to Tishby and Zaslavsky (2015) and Saxe *et al.* (2019), we compute the mutual information (MacKay, 2003) between a layer and the output predictions of the model. The idea is that if a layer is contributing substantially toward the prediction, it should have high mutual information with respect to the predicted labels. To this end, we compute the Adjusted Mutual Information (AMI) (Vinh *et al.*, 2010) between labels obtained by clustering the (hidden) representations of the samples at each of the layers and the labels predicted by the model. We apply two different clustering methods, K-means (Lloyd, 1982) and BIRCH (Zhang *et al.*, 1996). For reference, we compute the AMI also with respect to the true labels. Namely, Figure 8 shows how the AMI changes across layers for all the settings mentioned above.

All plots show a similar trend. In particular, the AMI is lower in the unconstrained block (reaching a minimum at the interpretable layer), and starts increasing in the monotonic block. This suggests that the monotonic block indeed plays a role in the classification, significantly raising the AMI score from its lowest value (interpretable layer) up to its maximum value (output layer). The plots for the AMI with respect to the true labels follow the same trend, although they are in general lower. This is expected since the classifier reaches ‘only’ 90% accuracy.

Note that if the monotonic block was not contributing to the classification (i.e. essentially behaving like an identity function), we would expect the AMI to remain constant from the interpretable layer to the output layer. This is because there is no information change through an identity function. Further note that if in the worst case, the monotonic constraint was hindering the classification (i.e. *losing* important information to separate the classes), we would instead observe a *decrease* of the AMI from the interpretable layer to the output layer.

#### 3.4.2 Activation across layers

A perhaps simpler way to understand where the classification happens is to look at the activation values of the neurons in each layer. An initial way of doing so is to simply visualize the mean activation values across layers stratified by cell type.

**Fig. 9.**
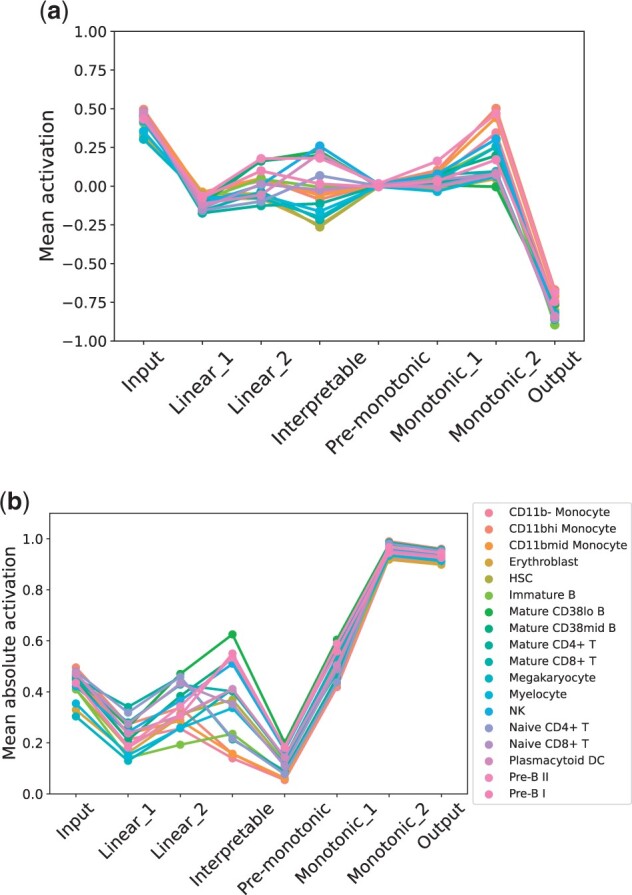
Mean activation (**a**) and mean absolute activation (**b**) of neurons across layers stratified by cell type. Both figures suggest that the unconstrained block reduces the variability of mean activation values, while the monotonic block has the effect of increasing drastically the mean absolute activation values

Figure 9 shows the mean activation of each layer stratified for each cell population. Both the mean activation (Fig. 9a) and the mean of the absolute value of the activation (Fig. 9b) are shown. From the plots, it seems the interpretable layer has the effect of reducing the variability of mean activation values across different cell types: Figure 9a shows that mean activation values are highly heterogeneous across cell types in the unconstrained block, and that this heterogeneity is reduced in the interpretable layer. The monotonic block has the effect of increasing mean activation values with an observable steep rise in absolute value. This can be a sign that the network is differentiating the classes more, as the activation values have to be substantially different from zero for the output layer to make a classification.

Further analyses supporting these claims are reported in Supplementary Section ‘More activation analyses’.

## 4 Conclusions

In this work, we propose a new class of structurally constrained deep models called MonoNets. Specifically, we impose a monotonic relationship between a layer of choice and the output/prediction layer. We argue that, in doing so, a user can achieve a higher level of transparency and interpretability. Since the monotonic relationship is enforced only on part of the network, the model is *not* fully interpretable with respect to the input features. We however argue that the goal of our proposed construction is to *increase* interpretability while attempting at *retaining as many approximation capabilities* of a neural network as possible.

We demonstrated our model on a cell-type classification task leveraging a single-cell proteomics dataset, as well as on benchmark risk prediction and image classification datasets. MonoNet shows promising results both in terms of accuracy and interpretability. An information-theoretic analysis of the monotonic block further shows that the constraints, we impose do not necessarily hinder the learning process of neural networks. It is however possible that in some prediction tasks, monotonicity may pose a too strong constraint. In these applications MonoNet may not be the best architecture to use: monotonicity is not *the* tool to use for interpretability but only *one of the possible ways to make neural networks more interpretable*.

In future work, we will explore the applicability of MonoNets to larger and more complex datasets. In particular, we will study more in-depth the effect of monotonic constraints on different deep models, e.g. deeper convolutional neural networks, recurrent neural networks (Rumelhart and McClelland, 1987) or transformers (Vaswani *et al.*, 2017). Another important research direction is to simplify the *full interpretability* of MonoNets. One possible solution is to identify the best *post hoc* methods to apply to the unconstrained block. An interesting idea in this direction is to leverage *interventional* causal methods, such as Brouillard *et al.* (2020). Indeed, since we are using causal methods to *post hoc* interpret a machine learning model, the model itself constitutes the data generating process. We are therefore able to generate interventional data. Alternatively, instead of relying on *post hoc* methods, we could use multiple monotonic blocks: by stacking consecutive monotonic blocks, we may be able to construct a *hierarchy of interpretable monotonic features* while retaining the learning capabilities of traditional neural networks.

## Supplementary Material

vbad016_Supplementary_Data
