# Supplementary material for: MonoNet: enhancing interpretability in neural networks via monotonic features
Source: Bioinform Adv. 2023 Feb 23;3(1):vbad016. doi: 10.1093/bioadv/vbad016 (PMC10152389; doi:10.1093/bioadv/vbad016)
Supplement: vbad016_Supplementary_Data [file vbad016_supplementary_data.pdf]

Supplementary

Extending MonoNets to other architectures

Convolutional Neural Networks (CNNs)

In Section "Interpretability analysis for colorectal cancer classification" we mentioned that to enforce monotonicity in a convolutional operation we just need to apply the constraint Eq. (2) to the weights of the convolution. Here we make this statement more precise. Since 2D convolutions are the most widely used, they will be the subject of this section. However, the same reasoning applies to convolutions of any dimensionality.

A 2D convolutional layer is composed of N convolutional filters. Each filter has a weight matrix $W \in \mathbb{R}^{H_f \times W_f \times C_f}$, where W_f, H_f, and C_f are, respectively, the width, height and number of channels of the filter. The convolutional layer will then output N feature maps of size depending on the size of the filter, the size of the input to the layer (including a possible padding), and the stride of the convolutional operation.[1]

Focusing of a single output feature map F, the values of the feature map in position (i, j) are given by

$$F(i,j) = \sum_{c=0}^{C_f-1} \sum_{h=0}^{H_f-1} \sum_{w=0}^{W_f-1} W(h,w,c) * I(i+h, j+w, c) \quad (6)$$

where I is the input to the convolutional layer, *e.g.* for the first convolutional layer I is typically the input image with $C_f = 3$ channels. From the above equation it is straightforward to see how to enforce monotonicity similarly to Eq.(2).

Convolutional layers are usually followed by an activation function, or pooling operations (*e.g.* average or max), which are monotonically increasing. Therefore, by enforcing monotonicity to Eq. (6), it is possible to obtain fully monotonic CNNs.

Recurrent Neural Networks (RNNs)

Recurrent Neural Networks (RNNs) [Elman, 1990] are neural network models designed to handle sequence/time-series data, *e.g.* "streaming" data that is presented at different time steps $t = 0, \ldots T$ up to a final step T. The basic building blocks of RNNs are recurrent units which, at time t, process the current input x_t, together with a hidden representation, h_{t-1}, which was recurrently updated up to the previous step $t-1$. The outputs are an updated version of the hidden representation h_t and, optionally, an output y_t. In the most basic formulation, a recurrent units processing has the following form:

$$\begin{aligned} h_t &= \sigma_h(W_h x_t + U_h h_{t-1} + b_h) \\ y_t &= \sigma_y(U_y h_t + b_y) \end{aligned} \quad (7)$$

where σ_h and σ_y are the typical activation functions. From this formulation, monotonicity can be easily enforced by constraining the matrices W_h, U_h, and U_y to be positive, similarly to Eq. (2).

A similar procedure can be done to enforce monotonicity in more complex recurrent units, such as LSTM [Hochreiter and Schmidhuber, 1997] and GRU [Cho et al., 2014] units. However, more care should be taken to ensure the monotonicity of the gating mechanism typical of these units.

[1] An example of computation of the output size is given in the reference documentation of the `pytorch` implementation at `https://pytorch.org/docs/stable/generated/torch.nn.Conv2d.html#torch.nn.Conv2d`

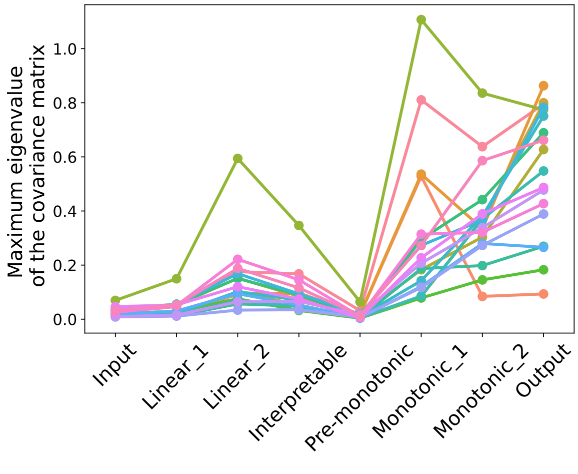

(a)

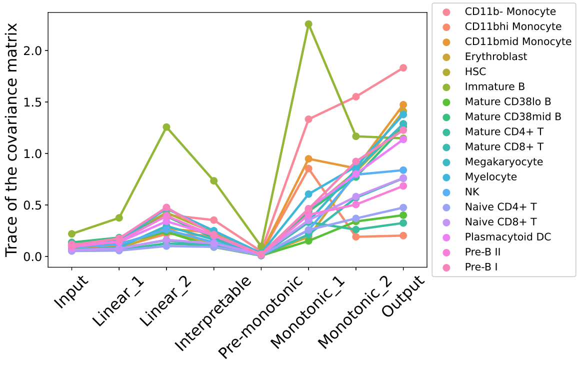

(b)

Fig. 10. (a) Maximum eigenvalue of the covariance matrix of the activation values stratified by layer and cellular type; and (b) trace of the same covariance matrices. Both Figures show that the variability in the activation values reaches a minimum in the interpretable layer before sharply increasing in the monotonic block. This is in agreement with Figure 9.

More activation analyses

One might wonder about the amount of heterogeneity in activation values for a given cellular type. To explore this, we compute the covariance matrix of the activation values within each layer for each cellular type. Figures 10a and 10b show, respectively, the maximum eigenvalue and the trace, i.e. the sum of the eigenvalues, of the computed covariance matrices. Similar patterns than those found when looking at the mean activation values are found. The interpretable layer has the lowest variability, while the variability substantially increases in the monotonic block. Both Figure 9 and Figure 10 support the idea of the interpretable layer acting as an information bottleneck [Tishby and Zaslavsky, 2015, Saxe et al., 2019] that substantially reduces the amount of information that is passed to the monotonic block, where the amount of information increases again. This is a way to look at the trade-off [Tishby and Zaslavsky, 2015] between compression (i.e. loss of information associated with the removal of non-informative patterns done on the unconstrained block) and classification accuracy in the monotonic block.

Supplementary figures and tables

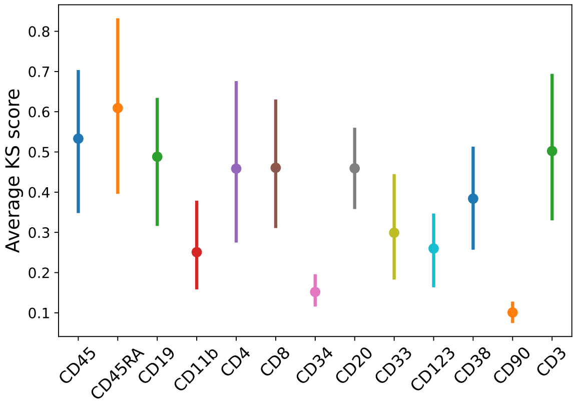

(a)

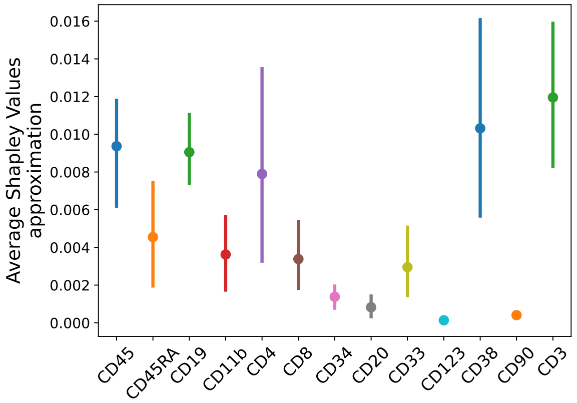

(b)

Fig. 11. Average of the KS scores (a) and Shapley values (b) across the interpretable neurons with the corresponding 95% confidence intervals. The Figures show a certain level of agreement between both strategies. For example, they both assign a high overall importance to markers CD3 and CD38, and low importance to markers CD90 and CD34

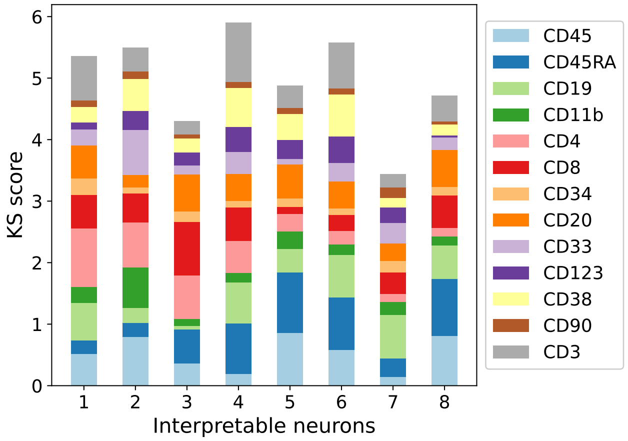

(a)

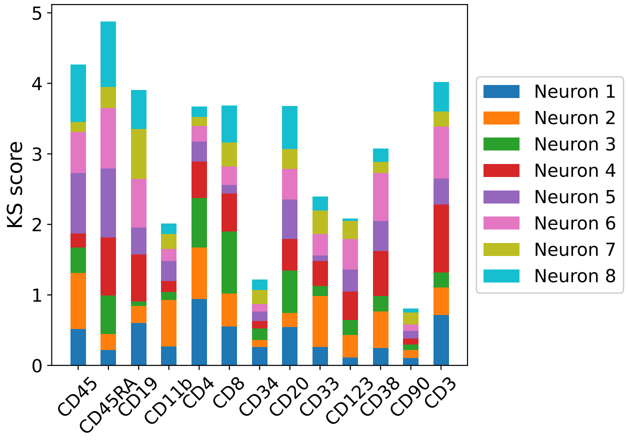

(b)

Fig. 12. KS scores between the top and bottom distributions of the input biomarkers ranked with respect to the activation values of the interpretable neurons. (a) shows the KS scores segregated by interpretable neuron and (b) by input biomarker.

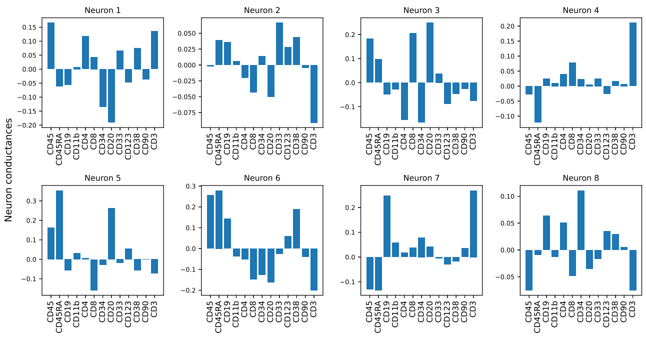

Fig. 13. Neuron conductances for all interpretable neurons and input biomarkers. Similarly to Figure 3, neurons in the interpretable layer show a specialization in the recognition of only a few markers.

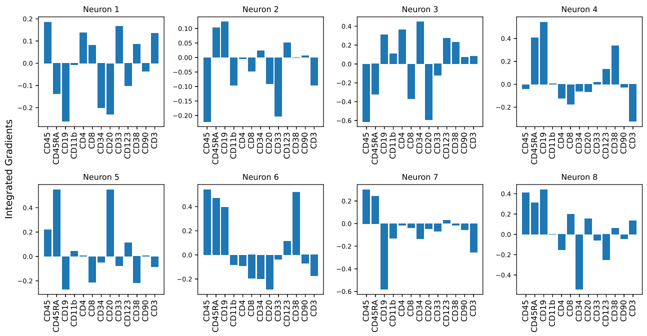

Fig. 14. Integrated Gradients for all interpretable neurons and input biomarkers. Similarly to Figure 3, neurons in the interpretable layer show a specialization in the recognition of only a few markers.

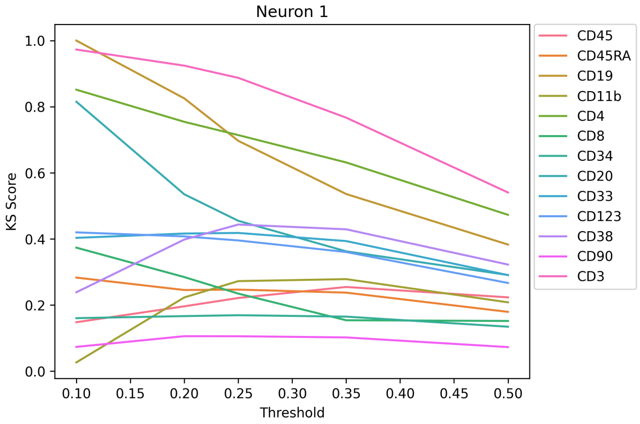

Fig. 15. KS Score vs. threshold chosen to describe bottom and top distribution in the statistical analysis described in paragraph (1) of Section "Interpreting the unconstrained block". The score is shown for each biomarker for the interpretable **Neuron 1**.

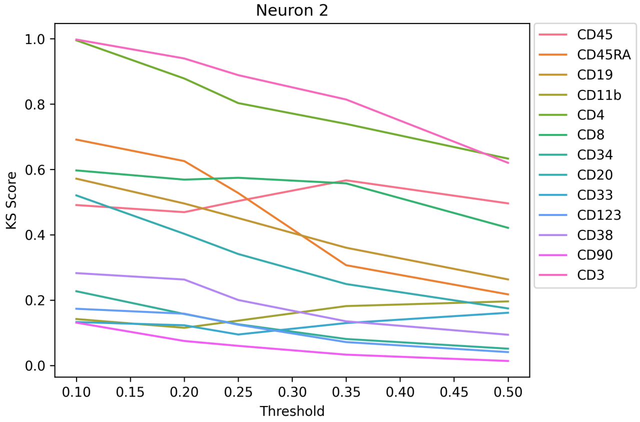

Fig. 16. KS Score vs. threshold chosen to describe bottom and top distribution in the statistical analysis described in paragraph (1) of Section "Interpreting the unconstrained block". The score is shown for each biomarker for the interpretable **Neuron 2**.

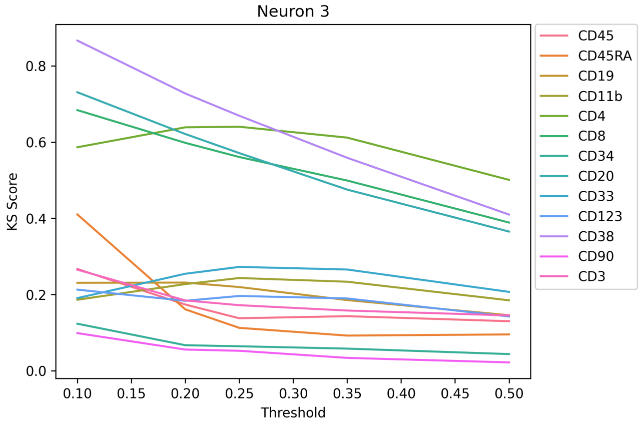

Fig. 17. KS Score vs. threshold chosen to describe bottom and top distribution in the statistical analysis described in paragraph (1) of Section "Interpreting the unconstrained block". The score is shown for each biomarker for the interpretable **Neuron 3**.

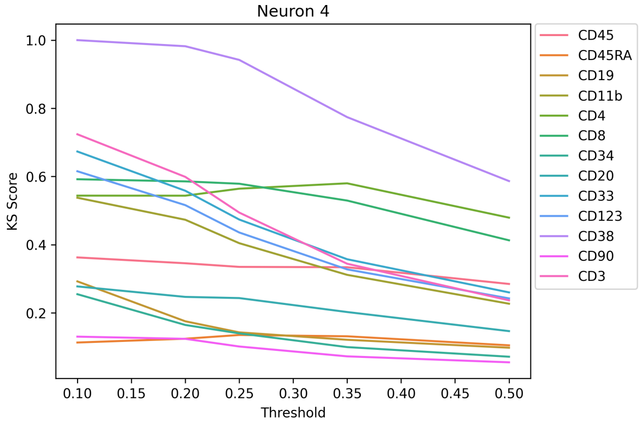

Fig. 18. KS Score vs. threshold chosen to describe bottom and top distribution in the statistical analysis described in paragraph (1) of Section "Interpreting the unconstrained block". The score is shown for each biomarker for the interpretable **Neuron 4**.

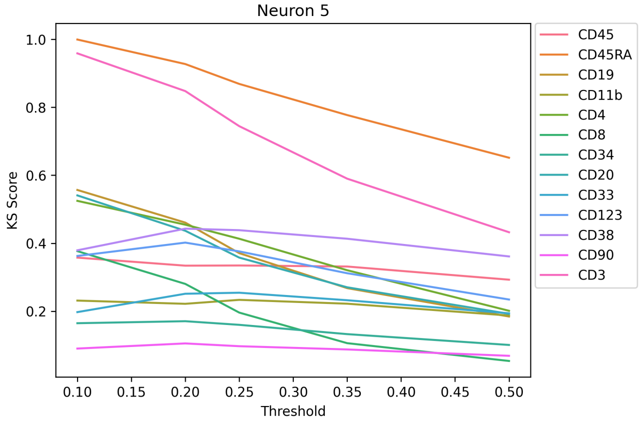

Fig. 19. KS Score vs. threshold chosen to describe bottom and top distribution in the statistical analysis described in paragraph (1) of Section "Interpreting the unconstrained block". The score is shown for each biomarker for the interpretable **Neuron 5**.

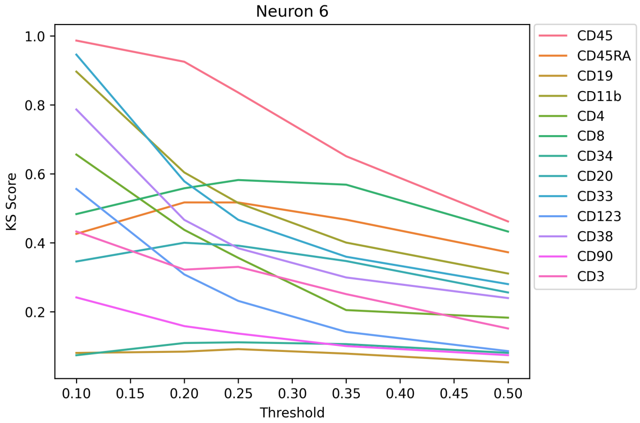

Fig. 20. KS Score vs. threshold chosen to describe bottom and top distribution in the statistical analysis described in paragraph (1) of Section "Interpreting the unconstrained block". The score is shown for each biomarker for the interpretable **Neuron 6**.

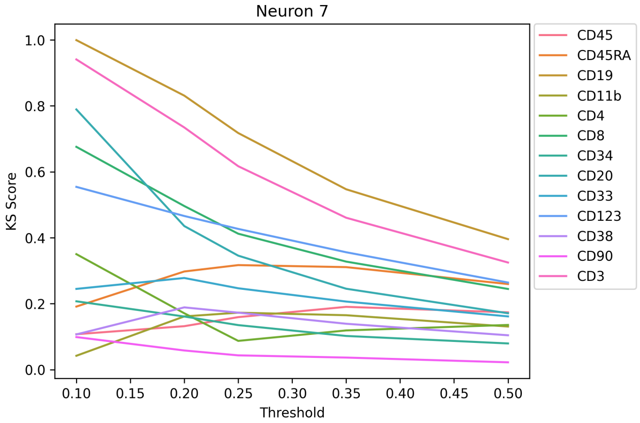

Fig. 21. KS Score vs. threshold chosen to describe bottom and top distribution in the statistical analysis described in paragraph (1) of Section "Interpreting the unconstrained block". The score is shown for each biomarker for the interpretable **Neuron 7**.

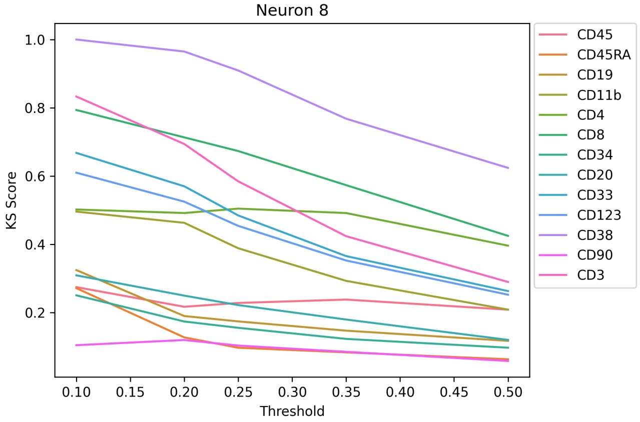

Fig. 22. KS Score vs. threshold chosen to describe bottom and top distribution in the statistical analysis described in paragraph (1) of Section "Interpreting the unconstrained block". The score is shown for each biomarker for the interpretable **Neuron 8**.

Table 3. Cellular populations present in the CyTOF dataset of Levine et al. [2015] with the markers characterizing each subpopulation.

	Classes	Biomarker Features
1	CD11b- Monocyte	CD33, CD11b
2	CD11b hi Monocyte	CD33, CD11b
3	CD11b mid Monocyte	CD33, CD11b
4	Erythroblast	CD45, CD38
5	HSC	CD45, CD34, CD38, CD45RA, CD90
6	Immature B	CD45, CD34, CD38, CD19, CD20, CD123
7	Mature CD38 lo B	CD45, CD34, CD38, CD19, CD20, CD123
8	Mature CD38 mid B	CD45, CD34, CD38, CD19, CD20, CD123
9	Mature CD4+ T	CD45, CD3, C19, CD33, CD4, CD8, CD45RA
10	Mature CD8+ T	CD45, CD3, C19, CD33, CD4, CD8, CD45RA
11	Megakaryocyte	
12	Myelocyte	CD123, CD33, CD11b
13	NK	CD45, CD45RA, CD38, CD3, CD19
14	Naive CD4+ T	CD45, CD3, CD19, CD33, CD4, CD8, CD45RA
15	Naive CD8+ T	CD45, CD3, CD19, CD33, CD4, CD8, CD45RA
16	Plasma Cell	CD45, CD19, CD20, CD38
17	Plasmacytoid DC	CD38, CD33, CD11b, CD123
18	Platelet	CD45, CD3, CD38
19	Pre-B II	CD45, CD34, CD38, CD19, CD20, CD123
20	Pre-b I	CD45, CD34, CD38, CD19, CD20

Table 4. Kolmogorov-Smirnov (KS) scores measuring the differences between the biomarker distributions of the top and bottom activating cells for all neurons of the interpretable layer and all markers.

	**CD45**	**CD45RA**	**CD19**	**CD11b**	**CD4**	**CD8**	**CD34**	**CD20**	**CD33**	**CD123**	**CD38**	**CD90**	**CD3**	**Average across biomarkers**
**Neuron 1**	0.518	0.217	0.606	0.267	0.944	0.552	0.261	0.544	0.259	0.114	0.250	0.105	0.720	0.41
**Neuron 2**	0.793	0.230	0.242	0.659	0.729	0.472	0.103	0.197	0.727	0.317	0.517	0.119	0.387	0.42
**Neuron 3**	0.368	0.546	0.059	0.118	0.701	0.873	0.163	0.610	0.144	0.214	0.220	0.072	0.215	0.33
**Neuron 4**	0.193	0.821	0.669	0.152	0.520	0.545	0.107	0.443	0.350	0.406	0.639	0.090	0.965	0.45
**Neuron 5**	0.857	0.984	0.381	0.288	0.280	0.118	0.134	0.559	0.082	0.312	0.422	0.101	0.362	0.38
**Neuron 6**	0.584	0.851	0.690	0.173	0.219	0.261	0.107	0.435	0.302	0.430	0.685	0.097	0.740	0.43
**Neuron 7**	0.142	0.301	0.710	0.207	0.133	0.344	0.195	0.284	0.333	0.253	0.153	0.173	0.212	0.26
**Neuron 8**	0.810	0.923	0.548	0.143	0.142	0.521	0.146	0.603	0.195	0.031	0.187	0.050	0.418	0.36
**Average across neurons**	0.54	0.67	0.47	0.25	0.39	0.45	0.14	0.45	0.30	0.28	0.40	0.10	0.47	
